# Shedding Light on Alzheimer’s β-Amyloidosis: Photosensitized Methylene Blue Inhibits Self-Assembly of β-Amyloid Peptides and Disintegrates Their Aggregates

**DOI:** 10.1038/s41598-017-07581-2

**Published:** 2017-08-08

**Authors:** Byung Il Lee, Yoon Seok Suh, You Jung Chung, Kweon Yu, Chan Beum Park

**Affiliations:** 1 KAIST Institute for the BioCentury, Department of Materials Science and Engineering, Korea Advanced Institute of Science and Technology (KAIST), Daejeon, Korea; 2 Neurophysiology & Metabolism Research Group, Korea Research Institute of Bioscience and Biotechnology (KRIBB), Daejeon, Korea; 3 Convergence Research Center for Dementia, Korea Institute of Science and Technology (KIST), Seoul, Korea; 4 Department of Functional Genomics, University of Science and Technology, Daejeon, Korea

## Abstract

Abnormal aggregation of β-amyloid (Aβ) peptides is a major hallmark of Alzheimer’s disease (AD). In spite of numerous attempts to prevent the β-amyloidosis, no effective drugs for treating AD have been developed to date. Among many candidate chemicals, methylene blue (MB) has proved its therapeutic potential for AD in a number of *in vitro* and *in vivo* studies; but the result of recent clinical trials performed with MB and its derivative was negative. Here, with the aid of multiple photochemical analyses, we first report that photoexcited MB molecules can block Aβ_42_ aggregation *in vitro*. Furthermore, our *in vivo* study using *Drosophila* AD model demonstrates that photoexcited MB is highly effective in suppressing synaptic toxicity, resulting in a reduced damage to the neuromuscular junction (NMJ), an enhanced locomotion, and decreased vacuole in the brain. The hindrance effect is attributed to Aβ_42_ oxidation by singlet oxygen (^1^O_2_) generated from photoexcited MB. Finally, we show that photoexcited MB possess a capability to disaggregate the pre-existing Aβ_42_ aggregates and reduce Aβ-induced cytotoxicity. Our work suggests that light illumination can provide an opportunity to boost the efficacies of MB toward photodynamic therapy of AD in future.

## Introduction

Methylene blue (MB) is a member of the phenothiazine family and has been utilized in pharmacology for more than a century. Since it was applied to malaria in 1891^1^, MB has been studied as a therapeutic agent for treating various diseases, proving its effectiveness against diseases such as methemoglobinemia and vasoplegic syndrome^2–4^. Owing to its ability to cross the blood-brain barrier (BBB) in addition to its high solubility in aqueous media and low toxicity, MB can target brain disorders in the central nervous system (CNS), such as ifosfamide-induced encephalopathy and Huntington’s disease, for which no effective cure exists yet^5, 6^. Recent studies reported that MB possesses a high potential for treating another common CNS disorder, Alzheimer’s disease (AD)^7^. MB was highlighted as a potential AD drug after TauRx Pharmaceuticals Ltd. presented successful results during phase II clinical trial performed with mild-to-moderate AD patients^8^. In addition, studies conducted with mouse AD models demonstrated that the treatment of MB not only reduces amyloid deposition but also improves behavior impairments including learning and memory defects by reducing amyloid plaque deposition in the brain^9, 10^. However, in spite of the encouraging results of the phase II clinical trial and *in vivo* studies performed with animal models, leuco-methylthioninium-bis(hydromethanesulfonate), a derivative of MB, failed to slow down the progression of AD in the phase III clinical trial, indicating a critical need for improved therapeutic options^11^.

**Figure 1:**
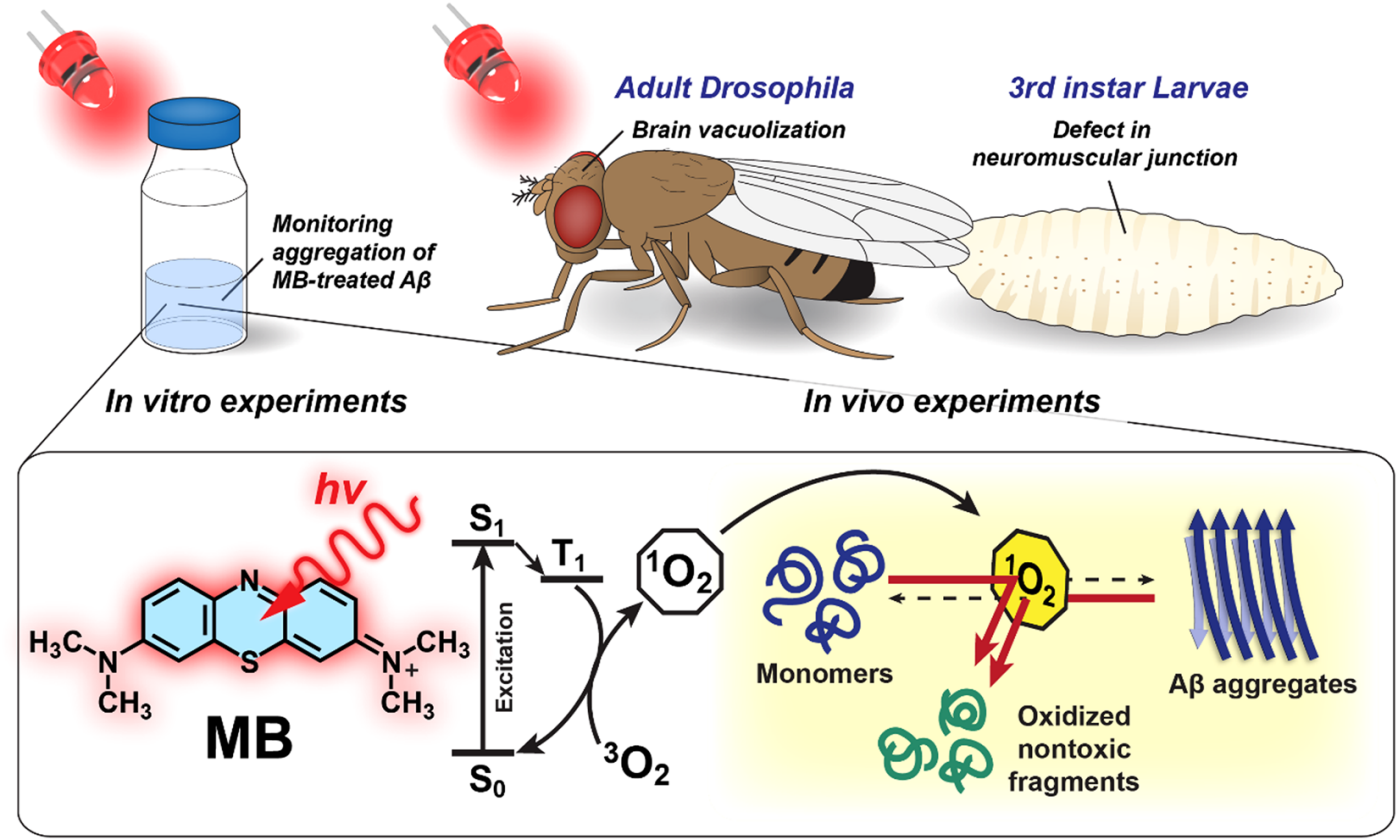
Schematic description of Aβ_42_ aggregation inhibition and the dissociation of pre-formed aggregates by photo-excited MB. The *in vitro* and *in vivo* experiments performed with the *Drosophila* AD model were conducted under the illumination of red LED light. The binding interaction of MB to Aβ_42_ aggregates and the photo-oxidation of the peptides induce disruption in the structural conformation, thereby blocking (or reversing) the progress of aggregation.

AD is the most prevalent neurodegenerative disease among people aged over 65, and the number of patients living with AD is growing in a high rate^12^. AD causes a gradual and irreversible decline in the patient’s cognitive ability and memory, which is characterized by abnormal accumulation of β-amyloid (Aβ) peptides of 39–43 amino acids^13^. Decades of studies have revealed that Aβ aggregation is a central pathological hallmark of AD, but the original function of Aβ and the mechanism by which Aβ self-assembly induces neurotoxicity have not been clearly elucidated^14^. Previous studies have shown that the aggregation of Aβ into β-sheet-rich oligomers or fibrils is a key pathogenic event in the onset of AD^15^. In this regard, the prevention of the self-assembly of Aβ monomers into aggregate states has been deemed vital for the treatment of AD. Over the years, researchers have made numerous efforts to screen small molecules that can inhibit Aβ aggregation^16^. Recently, photosensitizing chemicals have been explored for light-induced inhibition of Aβ assembly^17, 18^. For example, photosensitized riboflavin and water-soluble porphyrin molecules significantly suppressed Aβ aggregation by oxidizing the peptides in the early stage of Aβ assembly^17, 19^. MB is also known for its excellent photosensitizing property and has been extensively used for photodynamic treatment of cancer cells and microbes due to its high quantum yield of ^1^O_2_ generation (ϕ_Δ_ ~ 0.5) under red light^20, 21^. Based on the photochemical property of MB, here we explore light-induced inhibition of Aβ_42_ aggregation by MB *in vitro* as well as the suppression of synaptic toxicity in *Drosophila* AD model under light illumination, as depicted in Fig. 1. Furthermore, we investigated the possibility of disintegrating pre-formed Aβ_42_ aggregates by photo-excited MB molecules. One of the remarkable merits of MB as a photo-induced therapeutic agent for treating neurodegenerative diseases is its ability to cross BBB, which is regarded as a major difficulty for the development of brain-targeting drugs^22^. Furthermore, MB can be excited upon the absorption of red light (>630 nm), of which tissue penetration is better than that of green or blue light^23^. The higher tissue penetration depth of red light is a potential advantage of MB over previously reported, light-driven anti-amyloid aggregation agents of metal oxides and organic compounds, the absorption maxima of which lie at much lower wavelengths^17, 19, 24^.

The delivery of light into the brain tissue through the skull has been a major obstacle for the application of light in neuroscience and neuroengineering fields. Recent progresses in optogenetics, a technology to control a specific neural activity in the brain circuit using light^25^, facilitate the delivery of light to the target brain areas much feasible. To activate (or silence) a specific neural circuit, the researches illuminate the confined area using light guides such as fiber optics^26^. The optic fibers allows the light to be transferred to the deep brain areas, retaining its power density; they can easily be implanted in the head of freely moving animals. Moreover, recently development of wireless, implantable microLED platforms provide a minimal restriction in the behavior^27^. We envision that these recent advances in the implantable optoelectronic devices may lower the existing barrier in future applications of phototherapies to the neurodegenerative disorders.

## Results

### MB inhibits Aβ_42_ aggregation under light

**Figure 2:**
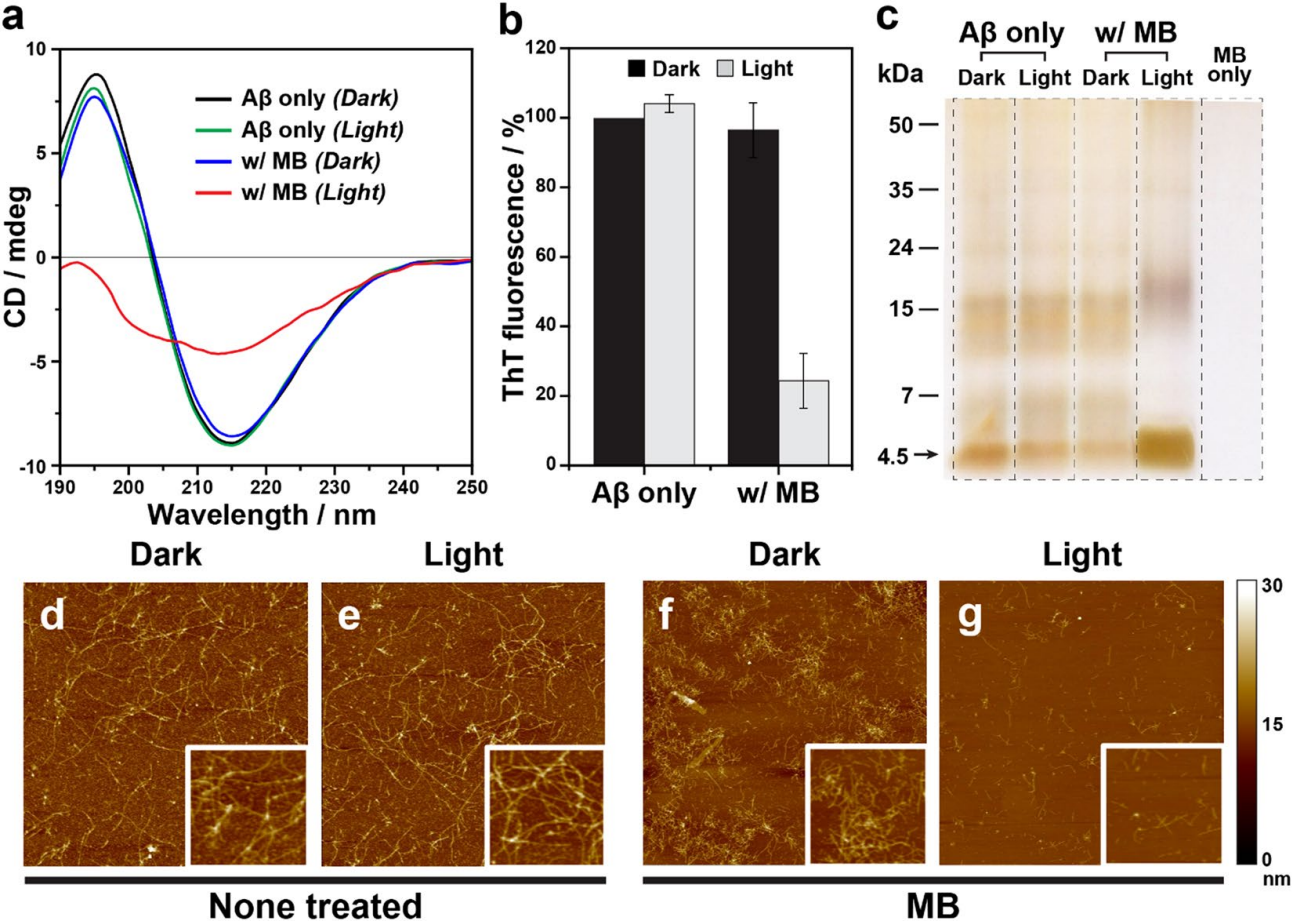
Light-induced suppression of Aβ_42_ self-assembly by MB. (**a**) CD spectra of Aβ_42_ aggregates incubated under various conditions. Two characteristic peaks in the CD spectrum presenting the β-sheet structure disappeared in the MB (10 μM)-treated Aβ_42_ (40 μM) under light illumination. (**b**) ThT fluorescence assay to measure the formation of amyloid fibrils. Significant decrease in ThT fluorescence indicates that the aggregation of Aβ_42_ monomers was substantially suppressed. (**c**) Silver-stained native gel electrophoresis showing that the monomeric contents was highly increased in MB-treated Aβ_42_ under light illumination. The arrow indicates a 4.5 kDa molecular mass that corresponds to the monomers of Aβ_42_. (**d–g**) Representative AFM images of Aβ_42_ incubated with or without MB under dark and light conditions. Only fragmented fibrils were observed in MB-treated Aβ_42_ (see insets enlarged from panels).

To observe the inhibitory effect of MB on Aβ_42_ aggregation under light, we performed multiple photochemical analyses. As shown in Fig. 2a, circular dichroism (CD) spectrum of Aβ_42_ incubated without MB under dark presented a positive and a negative band at 195 nm and 216 nm, respectively, which corresponds well to the typical profile of the β-sheet secondary structure. In the case of Aβ_42_ monomers (40 μM) incubated with MB (10 μM) under dark conditions, CD profile showed a negligible change, while the peaks completely disappeared in the presence of MB under light illumination. This result indicates that photosensitized MB molecules strongly affect the conversion of Aβ_42_ monomers into β-sheet rich aggregates. CD spectra of Aβ_42_ recorded at different times show that the secondary structure of the peptides are changed from random coil structure to β-sheet structure. (Figure S1) The diminished CD peaks monitored in Aβ_42_ treated with photo-excited MB implies that the unstructured Aβ_42_ monomers were remained after the incubation. Thioflavin T (ThT) fluorescence assay and atomic force microscope (AFM) analysis also support the photo-induced inhibitory effect of MB. MB-treated Aβ_42_ under dark conditions showed an insignificant decrease in ThT fluorescence compared to the native Aβ_42_ (Fig. 2b). While dense networks of mature fibrils were observed in the AFM images after incubation of Aβ_42_ monomers for 24 hours (Fig. 2d,e), numerous *short* Aβ_42_ fibrils were found when MB was treated (Fig. 2f). The short length of the fibrils is attributed to the accelerated rate of nucleation and fibril formation^28^. According to the previous study^29^, MB promotes the progress of Aβ_42_ fibrillation by stabilizing pre-nuclear intermediates that favor Aβ_42_ nucleation. In contrast, upon light illumination, substantially decreased ThT emission was monitored in the presence of MB, and only a limited number of aggregates were observed (Fig. 2g). The effect of photo-excited MB on the result of ThT assay was negligible according to the control experiment (Fig. S2). Native gel electrophoresis results revealed that the contents of Aβ_42_ monomers (4.5 kDa) increased significantly with photo-excited MB, implying that the considerable amount of monomers did not assemble into the aggregates of high molecular weight (Fig. 2c). Note that effect of MB on the reduction of Ag^+^ ion during the silver staining was negligible. The results obtained from sedimentation assay also demonstrate that the insoluble aggregates of Aβ_42_ were significantly reduced when the monomers were incubated with MB under light. (Figure S3) We further verified that the degree of photo-induced inhibition increased with the increasing MB concentration (Figs S4, S5). These photochemical analysis results clearly show that MB effectively suppressed the self-assembly of Aβ_42_ monomers into neurotoxic, β-sheet-rich aggregates under light. Further studies to investigate the effect of photo-excited MB on the oligomerization of Aβ_42_ or the equilibrium between various intermediates are needed. According to the literature, the distribution of Aβ_42_ oligomers at the certain time point can be assessed using photoinduced cross-linking of unmodified proteins (PICUP), which provides “snapshots” of the size distribution of various intermediates existing during the assembly^30^.

We further monitored the photo-induced Aβ_42_ aggregation inhibition effect by changing light wavelength, power density, and illumination time. We investigated the effect of the light wavelength using red (λ_max_ = 630 nm), green (λ_max_ = 520 nm), and blue (λ_max_ = 450 nm) LEDs. The maximum degree of inhibition was observed under red light, and the effect decreased with LEDs that had shorter light wavelengths (Fig. S6a). We attribute the result to the unique optical property of MB; as shown in Fig. S6b, the absorbance spectrum of MB overlaps mostly with the emission spectrum of red LED, but MB shows only a weak absorption in the shorter wavelength region (<550 nm). Moreover, we verified that the hindrance effect of photo-excited MB correlates with the power density of the light source (Fig. S7). In addition, when we shortened the illumination time from 24 h to 15 min, we could observe almost a similar degree of the inhibition effect on Aβ_42_ aggregation (Fig. S8), which indicates that light illumination for a very brief period is sufficient to induce a full capacity of photo-excited MB against Aβ_42_ aggregation. These results show that the efficacy of light-induced inhibition of MB can be easily controlled by the modulation of the light illumination system.

### Photo-excited MB suppresses Alzheimer’s Defects in *Drosophila*

**Figure 3:**
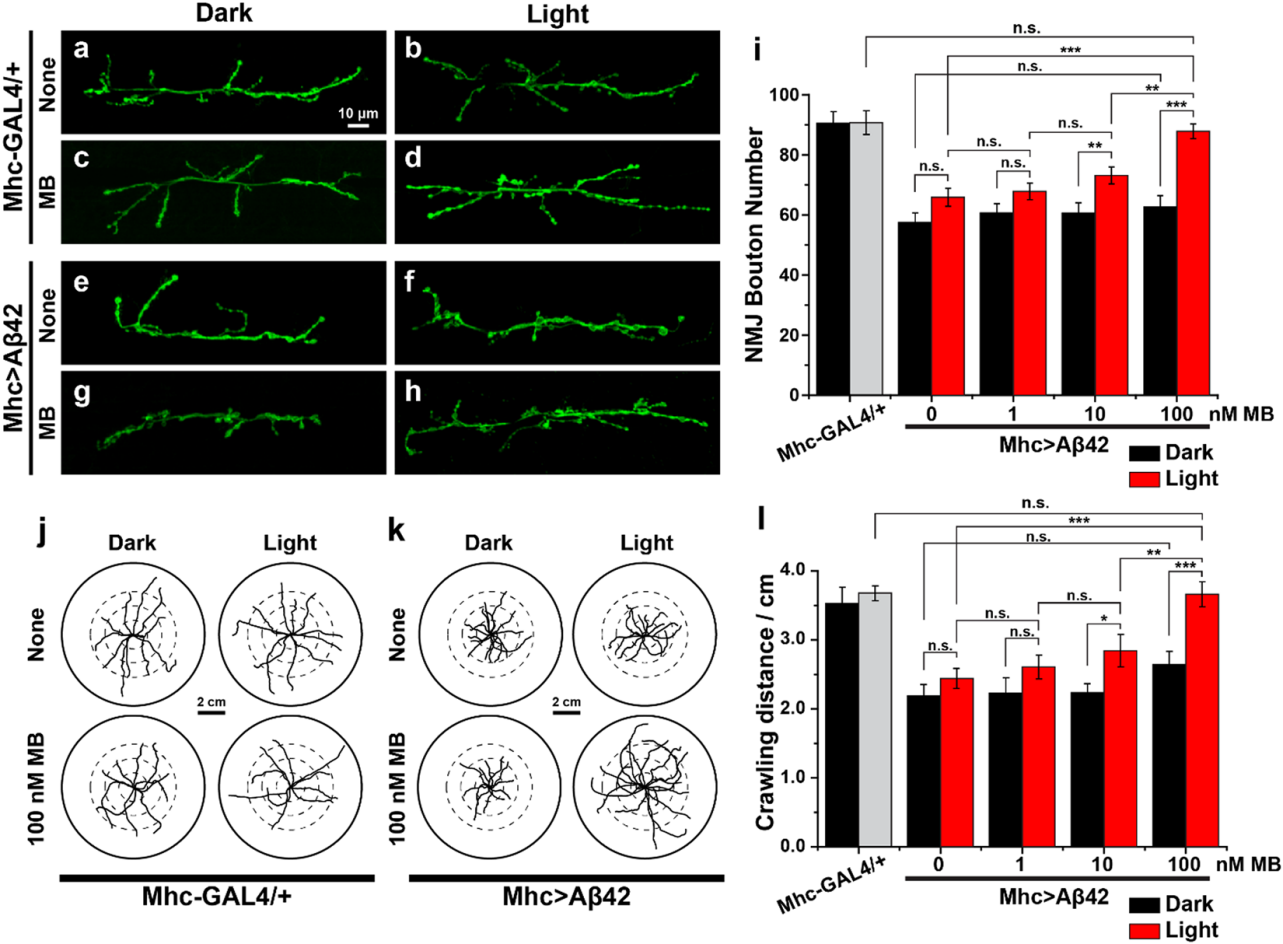
Photo-excited MB restores the phenotypes of Aβ_42_ toxicity *in vivo* model system. (**a–h**) The images of the NMJ boutons on muscle 6/7 of A3. Indicated genotype flies were incubated with or without 100 nM MB treatment under dark and red LED light. NMJ boutons were observed by HRP immunostaining. (**a**,**c**) NMJ of the *Mhc-GAL4/*+ control and (**e**,**g**) NMJ of the *Mhc > Aβ42* under dark condition; (**b**,**d**) NMJ of the *Mhc-GAL4/*+ control and (**f**,**h**) NMJ of the *Mhc > Aβ42* treated with or without 100 nM MB under red LED light. (**h**) NMJ morphology phenotype caused by Aβ_42_ overexpression is rescued by photoexcited-MB. Scale bar: 10 μm. (**i**) Effect of various concentration of MB on the total number of NMJ boutons on muscle 6/7 of A3. Indicated genotype flies were incubated with 0, 1, 10, and 100 nM concentration of MB under dark and red LED light conditions. (**j**,**k**) The diagram of the crawling path of the larvae on the plate. Diameter of inner-circles are 1.0 cm, 2.0 cm and 3.0 cm, respectively. (**j**) The crawling path of the *Mhc-GAL4/*+ control and (**k**) *Mhc > Aβ42* with or without 100 nM MB treatment under dark and red LED light. The locomotor phenotype in Aβ_42_ overexpression is rescued by photoexcited MB. Scale bar: 2 cm (**l**) Quantification of crawled distance of larvae within 90 seconds. Indicated genotype flies were incubated with 0, 1, 10, and 100 nM concentration of MB under dark and red LED light conditions.

**Figure 4:**
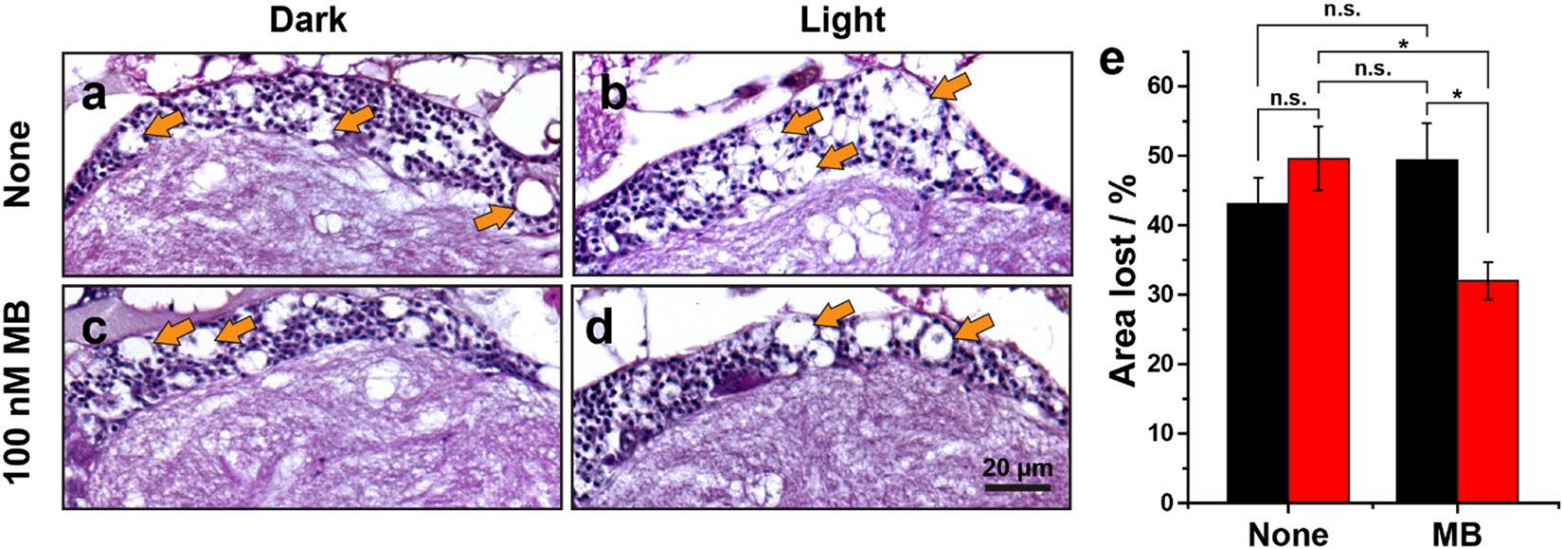
Photo-excited MB reduces the brain vacuolization in adult Drosophila. (**a**–**d**) Representative haematoxylin and eosin staining of adult head sections in AD model flies (*elav* > *Aβ42*) with or without 100 nM MB treatment under dark and red LED light conditions. Arrows indicate vacuole phenotypes in aged fly head. Scale bar: 20 μm. (**e**) Quantification of the vacuole size in adult head sections in AD model flies (*elav* > *Aβ42*) with or without MB treatment under dark and red LED light conditions. Percentage of the area lost in the cell body areas are shown as the averages s.e.m. (n = 5–7 hemispheres). The error bars represent means s.e.m. Experiments were performed at least three times. *P < 0.05, **P < 0.01, ***P < 0.001. n.s. not significant.

We further investigated the *in vivo* efficacy of photosensitized inhibition of Aβ_42_ aggregation by MB using *Drosophila* AD model. Animal models of human diseases are vital for understanding pathogenesis and for developing potential therapeutic agents. *Drosophila melanogaster* is one of most popular animal models due to its well-studied anatomical features that enable quantitative analysis of various phenotypes^31^. The AD model of *Drosophila* achieved by the overexpression of Aβ_42_ shows several neurodegeneration phenotypes, such as morphological defects of neuromuscular junction (NMJ), locomotion defects, and brain vacuolization^32, 33^. Here, we tested the suppression of the Aβ_42_-induced phenotypes by feeding MB to the *Drosophila* AD model under red LED light. The postsynaptic overexpression of Aβ_42_ (Mhc > Aβ42) leads to a significant loss in the synaptic bouton number^34^. We found that the number of bouton was reduced by ~30% in Mhc > Aβ42 larvae compared to the control (Mhc-GAL4/+) according to the confocal images of muscle 6/7 of abdominal segment 3 **(**Fig. 3a,e). A negligible improvement was monitored with the treatment of MB under dark or light illumination without MB. (Fig. 3f,g) The defect, however, was significantly rescued in the NMJ of larvae treated with 100 nM MB as well as light. (Fig. 3h,i) Reduced NMJ synaptic connection in the larvae is known to cause defective movement of the muscles related to the crawling behavior of the larvae and brain vacuolization^32, 35^. Figure 3j,k show that the larvae with postsynaptic overexpression of Aβ_42_ exhibited the reduction in crawling distance by more than 30% compared to the Mhc-GAL4/+ control. In contrast, when MB was fed to Mhc > Aβ42 larvae and was illuminated by red light, a significant improvement in the crawling behavior was observed in a dose-dependent manner (Fig. 3l), which coincides with the NMJ analysis results. When cultured under dark conditions, however, we could monitor only slight increase in the locomotion even when a highly concentrated MB was treated. We attribute the mild recovery of the phenotype to the therapeutic activity of MB itself without the aid of light, as reported previously^7, 9^. Yet, the photo-excited MB showed notably higher efficacy in a low dosage than static MB. We also found that photo-excited MB can reduce Aβ_42_ toxicity-induced brain vacuolization in the fly’s brain. In the brain of 30-days-old flies, overexpression of Aβ_42_ (elav > Aβ42) showed severe brain vacuolization in the cell body region compared to the elav-GAL4 control (Figs 4a, and  S9). In contrast, the number of vacuoles of photo-excited-MB-treated elav > Aβ42 was reduced compared to that of non-MB-treated elav > Aβ42 fly brains. The lost area of the photo-excited-MB-treated elav > Aβ42 fly brains were reduced by ~20% compared to non-treated flies (Fig. 4b). This significant restoration of Aβ_42_-induced toxicity in the elav > Aβ42 fly brains coincided with the results of the locomotion defect experiment. Taken together, these results suggest that photo-excited MB can suppress defects of NMJ morphology, locomotion defects, and Aβ_42_-induced toxicity in the *Drosophila* AD model.

### Photo-excited MB dissociate the pre-exisiting aggregates

**Figure 5:**
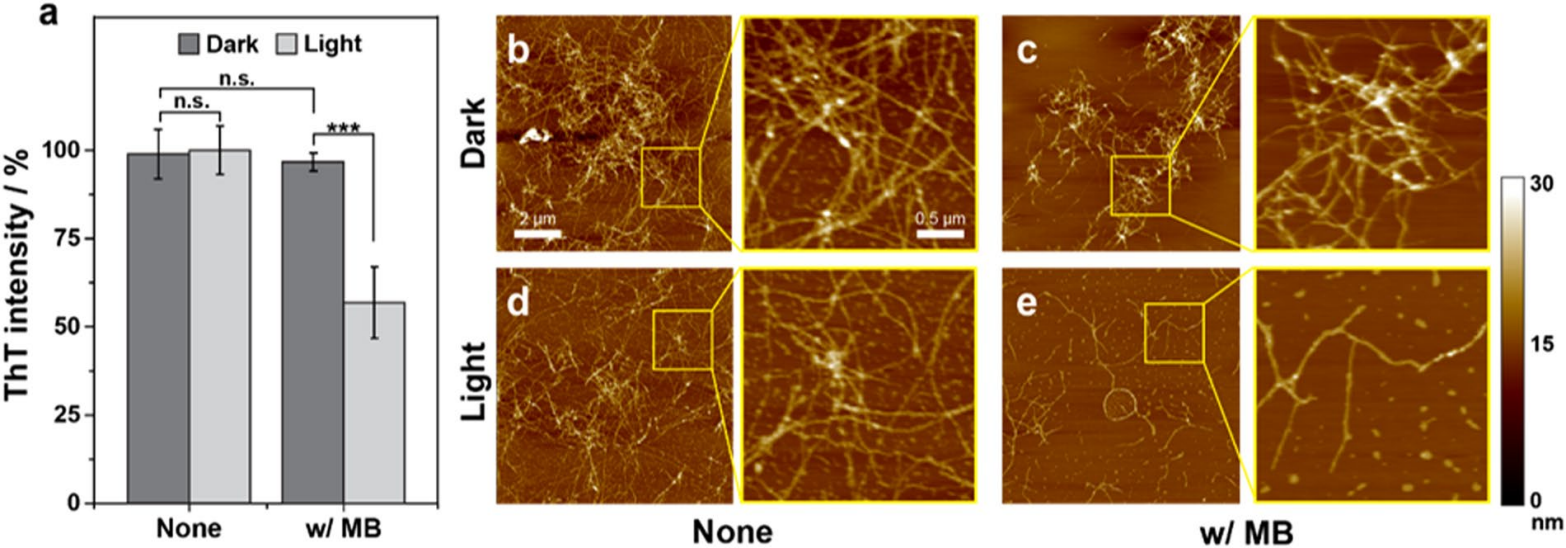
Disassembly of pre-formed Aβ_42_ aggregates by photoexcited MB (10 μM). The fully-grown Aβ_42_ aggregates were formed by the incubation of monomeric Aβ_42_ for 48 hrs at 30 °C. (**a**) ThT fluorescence of Aβ_42_ aggregates after the 6 hrs of treatment with MB under dark and light conditions. (**b**–**e**) AFM images of fully-grown Aβ_42_ aggregates after 6 hrs of incubation in the absence or presence of 10 μM MB under dark and light conditions. The fully-grown aggregates were produced by incubation of Aβ_42_ monomers for 42 h at 30 °C.

While numerous studies have focused on the inhibition of the Aβ_42_ assembly pathway, recent studies have attempted to reverse the progress by dissociating pre-formed Aβ_42_ aggregates^36–38^. Previous studies have demonstrated that the clearance of pre-existing amyloid deposits could reverse AD pathology, including behavioral deficits, in transgenic mouse models^36, 39^. To examine the possibility of disassembling Aβ_42_ aggregates by photo-excited MB, we incubated pre-formed aggregates with MB under dark or light conditions and monitored the changes in ThT fluorescence, morphology, and cytotoxicity. For the experiment, Aβ_42_ monomers were incubated for 48 h to produce fully-grown, fibrillar aggregates. According to the ThT assay result (Fig. 5a), ThT fluorescence was drastically diminished (~50%) when photo-excited MB was applied, while MB under dark conditions caused a negligible decrease. The corresponding AFM images also confirmed that the density of the fibril networks decreased in the presence of photo-excited MB (Fig. 5f). Both the results of the ThT assay and the AFM images clearly indicate that light triggers disassembly of existing Aβ_42_ aggregates when incubated with MB. Additional researches such as size-exclusion chromatography (SEC) and *in vivo* studies using the brain of mouse AD models are required to further investigate the efficacy of photo-excited MB against pre-formed aggregates.

## Discussion

**Figure 6:**
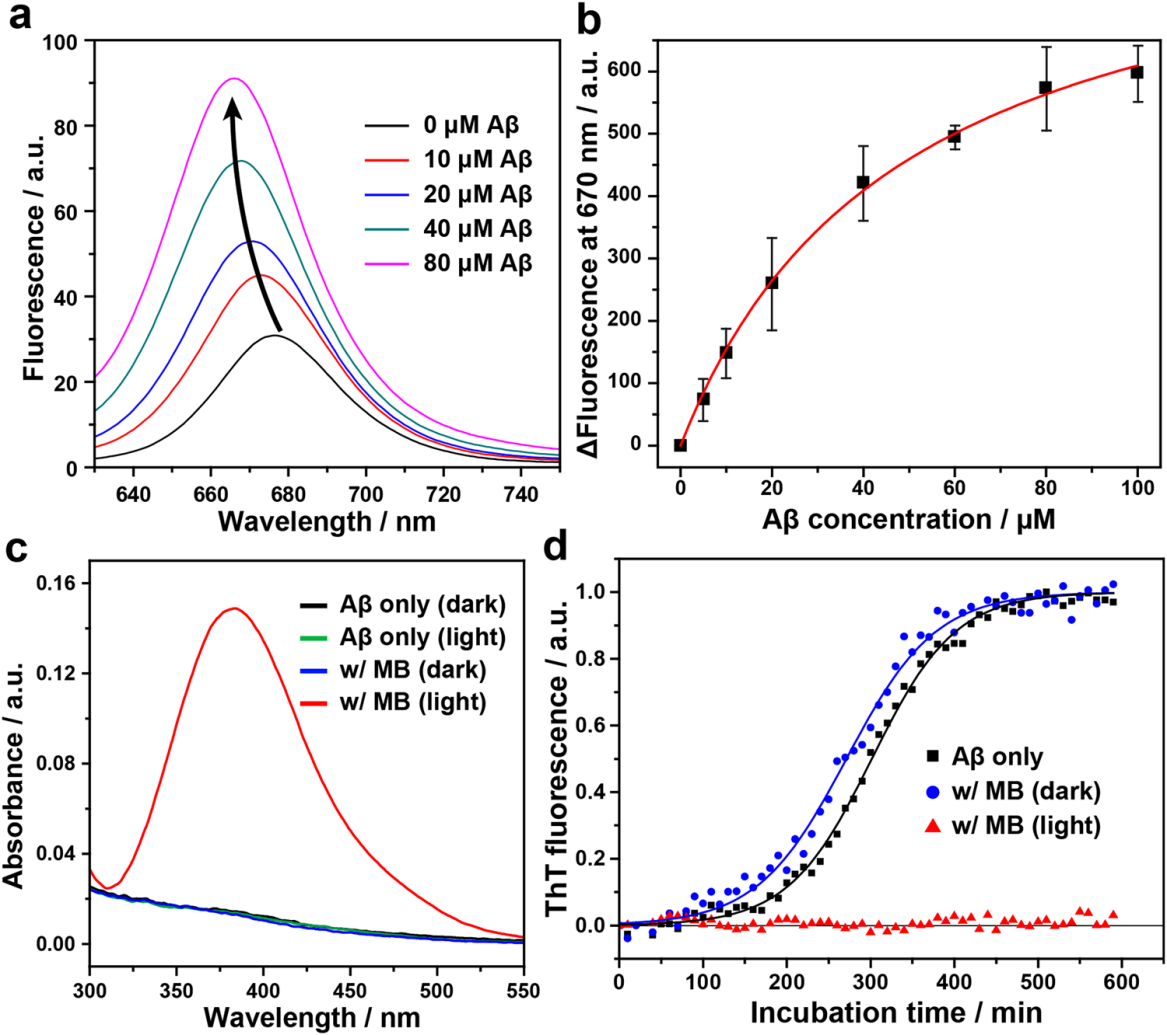
Photo-excited MB inhibits the Aβ_42_ aggregation by means of the binding affinity of MB and the photo-oxidation of Aβ_42_. (**a**) Change in fluorescence spectra of 0.2 μM MB upon addition of various concentrations of the Aβ_42_ monomer. (**b**) Fluorescence binding affinity assay between Aβ_42_ and MB. The fluorescence of MB (0.2 μM) at 670 nm was measured with increasing concentration of Aβ_42_ from 0 to 100 μM. Binding constant K_d_ was derived from the fitted curve. (**c**) DNPH assay to monitor a carbonyl content in the Aβ_42_ peptide. DNPH reacts with the carbonyl groups in oxidized peptides, resulting in the formation of a DNP hydrazone product, which shows an absorption maximum of near 380 nm. (**d**) The kinetics of Aβ_42_ fibril formation monitored at 30 °C by ThT fluorescence in the absence and presence of MB under dark or light conditions. For the light condition, the MB-treated Aβ_42_ samples were irradiated with LED light for 30 min at 4 °C before the measurement. Each point is an average of the fluorescence signal of at least four wells containing the same solutions. Lines indicate fits of a sigmoidal growth curve.

We attribute the light-induced hindrance effect of MB to its high binding affinity to Aβ_42_ and oxidative stress generated from photochemical reactions. To investigate the interaction between MB and Aβ_42_, we monitored the change of MB fluorescence in the presence of Aβ_42_. We observed enhanced fluorescence of MB with a blue shift with an increasing concentration of Aβ_42_ peptides (Fig. 6a). According to the literature^40^, the fluorescence enhancement can be attributed to the reduction in the non-radiative decay of photo-excited MB due to the suppressed rotation and vibration upon binding to other chemicals. The blue shift of the fluorescence of fluorophore occurs when a dye exists in a more non-polar environment because the energy difference between the excited and ground state increases^41, 42^. This implies that MB may bind to the hydrophobic C terminus of Aβ_42_ monomers^43^. Further studies, including computational simulations, are required to predict the exact binding site of MB to Aβ_42_. The binding constant (*K*_d_) of MB to Aβ_42_, estimated from the changes in the fluorescence intensity at 670 nm for various MB/Aβ_42_ ratios, was 48.7 ± 3.6 μM (Fig. 6b). This is comparable to the *K*_d_ of curcumin, a well-known small molecular inhibitor of Aβ_42_ aggregation (*K*_d_ = 46 μM)^44^.

The capacity of MB as a light-driven ^1^O_2_ generator has been widely utilized in a number of studies^45, 46^. Under the irradiation of red light (λ_max_ = 666 nm), MB monomers produce ^1^O_2_ through the type II photochemical pathway, in which the energy from triplet state MB (i.e., ^3^MB^+^) is transferred to molecular oxygen^20^, and the generated ^1^O_2_ oxidizes organic compounds nearby^47^. To explore the possible photo-oxidation of Aβ_42_ by MB, we conducted 2,4-dinitrophenylhydrazine (DNPH) assay, which is one of the most commonly used methods to assess the amount of carbonyl groups formed by oxidative stress^48–50^. As shown in Fig. 6c, we observed a new absorption band at 380 nm only when Aβ_42_ was incubated with MB under light illumination, indicating that Aβ_42_ peptides were oxidized by photo-excited MB. In addition, our ThT assay and CD analysis revealed that the hindrance effect of photo-excited MB on Aβ_42_ aggregation decreases significantly under anaerobic conditions (Figs S10, S11). These results indicate that the generation of ^1^O_2_ is a major cause of light-induced inhibition of Aβ_42_ assembly by MB. We suppose that the generated oxidative stress induces sulfoxidation of Aβ_42_’s methionine, which is known to be a most readily oxidizable residue^51^. According to the literature^52^, the oxidation of Met35 causes structural alteration in the hydrophobic C-terminus of Aβ_42_ and impedes the association and self-assembly between monomers. The oxidation of Aβ_42_ by photo-excited MB was further studied with matrix-assisted laser desorption ionization time-of-flight mass spectrometry (MALDI-TOF MS). Figure S12 shows that the mass of the MB-treated Aβ_42_ increases when the light was irradiated. We attribute a +14 Da-modification to the oxidation of His13 or His 14 residues, which generates a dehydro-2-imidazolone derivative^53^. According to the literature, the further increases in the mass of Aβ_42_ by 16 Da are resulted from the oxidation of Met35 and Tyr10^19^. We monitored how static and photo-excited MB affect the aggregation kinetics of Aβ_42_ differentially using ThT fluorescence assay. For the experiment, we pre-incubated MB-treated Aβ_42_ solution for 30 min at 4 °C under dark or light conditions before the measurement was performed at 30 °C. According to our results (Fig. 6d), while static MB slightly affected the nucleation of Aβ_42_ peptides with a decreased lag time from 202.0 min to 164.7 min, photo-excited MB completely blocked the progression of Aβ_42_ aggregation in the early stage. We attribute the hindered aggregation to the oxidation of Aβ_42_ monomers by localized ^1^O_2_ generated during light illumination. The negligible inhibitory activity of pre-illuminated MB supports that the hindrance effect of photo-excited MB was derived from the photodynamic reaction of MB under light. (Figure S13 **)** For the therapeutic applications, it is vital to minimize the undesirable oxidative damages to the surrounding by limiting the ^1^O_2_ generation sites. Future studies to enhance the Aβ-specific targeting of photosensitizers are essential for the phototherapy of amyloidosis.

In summary, we demonstrated that photo-excited MB molecules exhibit a high degree of inhibition against β-amyloidosis *in vitro* and *in vivo*. Our kinetic study revealed that, while static MB accelerates Aβ_42_ aggregation, MB under illumination thoroughly blocks the progress in the early stage by oxidizing the peptide. We examined the *in vivo* effect of light-induced inhibition of aggregation by MB using the *Drosophila* AD model. At the same dose, while static MB exhibited mild recovery in the locomotion defect, photoexcited MB almost fully rescued the AD phenotype in *in vivo* experiments performed with the *Drosophila* AD model; the loss in synaptic bouton, locomotion defect, and vacuolization in the brain were significantly reduced with the MB treatment under red LED light, indicating that photoexcited MB successfully prevented *in vivo* toxicity resulting from β-amyloidosis. We further verified that MB under illumination is also able to dissociate pre-existing aggregates and to suppress resulting cytotoxicity, while MB under dark conditions did not affect the aggregation state. Based on these results, we suggest that shining light on MB can be a breakthrough to enhance its efficacy beyond the conventional limit. While the recent report on clinical trials performed with MB was not satisfactory, this study hints at a new opportunity of inhibiting β-amyloidosis based on the photosensitizing property of MB, a therapeutic chemical that has been used for more than a century.

## Methods

### Preparation of Aβ_42_ Peptides

Recombinant β-amyloid (1–42) was purchased from rPeptide Co. Monomeric Aβ_42_ solution was prepared by dissolving the peptide in hexafluoro-2-propanol (HFIP) followed by sonication for 30 min and keeping it overnight at room temperature. The solution was aliquoted into 1.5 ml protein LoBind tubes (Eppendorf) and vacuum-dried for 2~3 h. The tubes were then stored at −20 °C for further use.

### Light-induced inhibition of Aβ_42_ self-assembly

Aβ_42_ aliquot was dissolved in a 30 μL buffer comprised of CH_3_CN (144 μM), Na_2_CO_3_ (144 μM) and NaOH (8.5 mM) and was briefly sonicated for 1 min. The solution was diluted with 270 μL of phosphate buffer (8.5 mM) containing NaCl (8.5 mM), Na_2_CO_3_ (14 μM), NaOH (0.85 mM), and 6.0% acetonitrile (final pH 8.0) to yield a final concentration of 40 μM Aβ_42_ monomer. For the *in vitro* experiments, the solution was incubated in the absence or presence of methylene blue (MB) at 30 °C for 24 h under dark or light conditions. The power densities of the light sources (LEDs) were measured with ILT1400-A photometer (International Light Tech.). MB and all other chemicals were purchase from Sigma Aldrich.

### Disassembly of pre-formed fibrils

The pre-formed Aβ_42_ aggregates were obtained by the incubation of the Aβ_42_ monomers (40 μM) for 48 h at 30 °C. Then, 10 μM MB was added to the solution containing aggregates, and further incubated for an additional 6 h under dark or light conditions at 30 °C.

### Circular dichrosim (CD) analysis

After the incubation of 40 μM Aβ_42_ under various conditions at 30 °C for 24 h, far-UV (190–260 nm) CD spectra were measured using a JASCO J-810 (Jasco) spectropolarimeter at 20 °C.

### Thioflavin T (ThT) assay

The 20 μl of incubated Aβ_42_ samples were mixed into 480 μl of ThT solution (20 μM) in the phosphate buffer. The fluorescence of ThT was measured at 440 nm (ex) and 485 nm (em) using RF-5301PC spectrofluorophotometer (Shimadzu Inc.). For the real-time monitor of Aβ_42_ aggregation, monomeric Aβ_42_ (5 μM) in the absence or presence of 2 μM MB in a glass vial was incubated at 4 °C for 30 min under dark or light conditions, prior to the measurement. Then, 90 μl of samples were moved to a 96-well plate, and 10 μl of ThT solution (final concentration, 20 μM) was added to each well. The fluorescence of ThT was monitored every 10 min using a 405 nm excitation filter and a 486 nm emission filter of the Victor 3 microplate reader (PerkinElmer Inc.). The temperature of the well plate was maintained at 30 °C during the measurement. Each experimental point was an average of the fluorescence signal of at least four wells containing the same solution.

### Atomic Force Microscopy (AFM)

For the AFM measurement, 5 μl of Aβ_42_ sample solutions were deposited onto a cleaved mica substrate for 10 min and were rinsed several times with DI water to remove remaining salts and unbound peptides. After they were fully dried, AFM images were acquired in a tapping mode with an NCHR silicon cantilever (Nanosensors Inc.) using a Multimode AFM instrument equipped with a Nanoscope III controller and “E”-type scanner (Digital Instruments Inc.).

### Native gel electrophoresis and silver staining

The Aβ_42_ solutions were transferred to a loading buffer containing 50 mM Tris HCl, pH 6.8, 1% SDS, 1% β-mercaptoethanol, 10% (v/v) glycerol, and 0.01% bromophenol blue. The samples were loaded onto 10% Gradi-Gel™ II gradient gel (Elpis Biotech) and peptide distribution was visualized by silver staining. Protein electrophoresis kit were purchased from Bio-rad.

### Sedimentation assay

The sedimentation assay was performed according to the previous study^54^. Briefly, Aβ_42_ monomers (40 μM) were centrifuged at 10,000 g for 10 min at 4 °C. The supernatant was collected and the optical density (OD) at 214 nm was measured using V/650 spectrophotometer (Jasco Inc.). The supernatant was then moved to the glass vials and MB (2 μM) was introduced to the vials. The samples were incubated under dark or light conditions for 24 h at 30 °C. Then the samples were ultracentrifuged at 100,000 g for 10 min at 4 °C. The OD_214_ of collected supernatant was measured. The aggregation was derived from the difference between the OD_214_ before and after the incubation as described by Yoshiike *et al*.

### DNPH assay

The DNPH assay was performed according to Dalle-Donne *et al* ^48^. Aβ_42_ solutions (40 μM) with or without 10 μM MB were incubated under dark or light conditions for 24 h at 30 °C. The samples were contained in the glass vials and illuminated with red LED (λ_max_ = 630 nm, 3 mW/cm^2^) The samples were then precipitated with a trichloroacetic acid (TCA, 20% final concentration) solution for 10 min in an ice bath and were then collected by centrifuge. Then, 2 M HCl containing 10 mM DNPH (2 M HCl only for reagent blanks) was added and incubated for 1 h under room temperature. After the precipitation with 20% TCA and centrifugation, the remaining pellets were washed three times with 1 ml ethanol-ehtyl acetate (1:1, v/v) solution. The samples were then dissolved in 6 M guanidine hydrochloride solution (in 20 mM potassium phosphate, pH 2.3 adjusted with TCA) and were incubated at 37 °C for 15 min. The absorbance spectrums of the samples were measured using V/650 spectrophotometer (Jasco Inc.).

### Mass spectrometry

Aβ_42_ monomer (40 μM) was treated with MB (1 μM) and irradiated for 0, 0.5, 1, 2, and 3 h at 4 °C. MALDI-TOF spectra was recorded with Bruker autoflex III (Bruker Daltonics) using sinapinic acid as a matrix. 1.5 μl of samples mixed with matrix (1:1) was spotted on the plate.

### Fly strains

The UAS-Aβ42 was provided by Dr. K Iijima-Ando^55^, the Mhc-GAL4 driver was provided by Dr. T Littleton; and the elav-GAL4 driver was provided by Bloomington Stock Center. For the pharmacological approach, either MB or PBS was added to fly food at 1, 10, 100, 1000, and 10000 nM concentration. All flies were reared at 25 °C.

### Brain vacuole analysis

For analysis of brain vacuolization, 30-days-aged fly heads were fixed in 4% paraformaldehyde (Electron Microscopy Sciences) and were processed for paraffin sections as described^32^. Embedded paraffin was cut into 4 um-thick coronal sections. These sections were stained with hematoxylin and eosin (Vector laboratories). For quantification of vacuole phenotypes in the fly head section, we measured the area of the vacuoles in the cell body region using ImageJ. Five to ten hemispheres were analyzed for each genotype. For the pharmacological approach of the brain vacuole analysis, either MB or PBS was added to fly food at 100 nM concentration.

### Immunohistochemistry

Third instar larvae were dissected in PBS, fixed in 4% formaldehyde (Ted Pella) in PBS for about 15 minutes and washed 3x in 0.1% Triton X-100 in PBS. FITC-conjugated anti-HRP (Jackson ImmunoResearch Laboratories) were used at 1:100 and were incubated for about 1.5 hours at room temperature. Laval samples were mounted in SlowFade Antifade kit (Invitrogen). Confocal images were collected from OLYMPUS FLUOVIEW FV-1000 confocal microscopes SP2 equipped with 40x UPlans FL N inverted oil lens. OLYMPUS Fluoview software was used to capture, process, and analyze the images. Analysis of the NMJ was performed essentially as described^34^.

### Crawling assay

Wandering 3rd instar larvae were briefly washed with PBS to remove residual fly food. Larvae were dried for a short time on clean filter paper and were placed on a 2% agar-grape juice coated petri dish. Each genotype was allowed to crawl freely for 90 sec. To quantify the crawling distance, we drew lines to track crawled larvae and measured the distance using Image J software. Approximately 10–20 animals were tested for each genotype.

## Electronic supplementary material

Supporting Information
