# Supplementary material for: Shedding Light on Alzheimer’s β-Amyloidosis: Photosensitized Methylene Blue Inhibits Self-Assembly of β-Amyloid Peptides and Disintegrates Their Aggregates
Source: Sci Rep. 2017 Aug 8;7:7523. doi: 10.1038/s41598-017-07581-2 (PMC5548810; doi:10.1038/s41598-017-07581-2)
Supplement: Supplementary file 1 — Supporting Information [file 41598_2017_7581_MOESM1_ESM.doc]

Supplementary Information

**Shedding Light on Alzheimer's β-Amyloidosis: Photosensitized Methylene Blue Inhibits Self-Assembly of β-Amyloid Peptides and Disintegrates Their Aggregates**

Byung Il Lee^1,†^, Yoon Seok Suh^2,3†^, You Jung Chung^1^, Kweon Yu^2,3,4^, and Chan Beum Park^1,*^

^1^KAIST Institute for the BioCentury, Department of Materials Science and Engineering, Korea Advanced Institute of Science and Technology (KAIST), Daejeon, Korea. ^2^Neurophysiology & Metabolism Research Group, Korea Research Institute of Bioscience and Biotechnology (KRIBB), Daejeon, Korea. ^3^Convergence Research Center for Dementia, Korea Institute of Science and Technology (KIST), Seoul, Korea. ^4^ Department of Functional Genomics, University of Science and Technology, Daejeon, Korea.

**
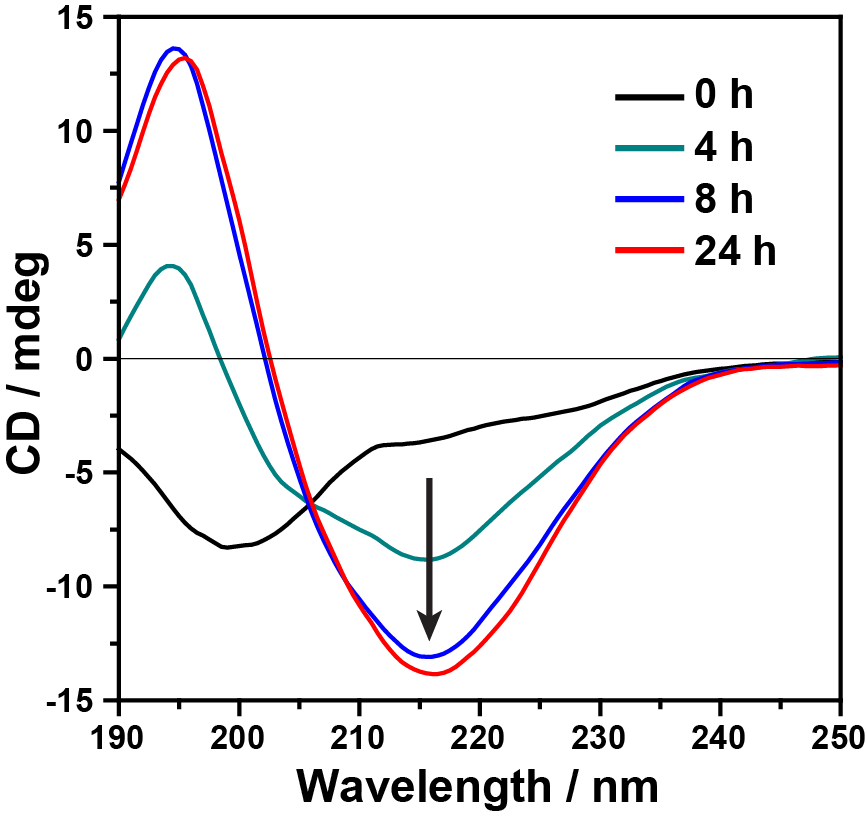
**

**Figure S1.** CD spectra showing the effect of incubation time on Aβ_42_ aggregation. Aβ_42_ (40 μM) solution in a phosphate buffer was incubated at 30˚C for 0, 4, 8, and 24 h.


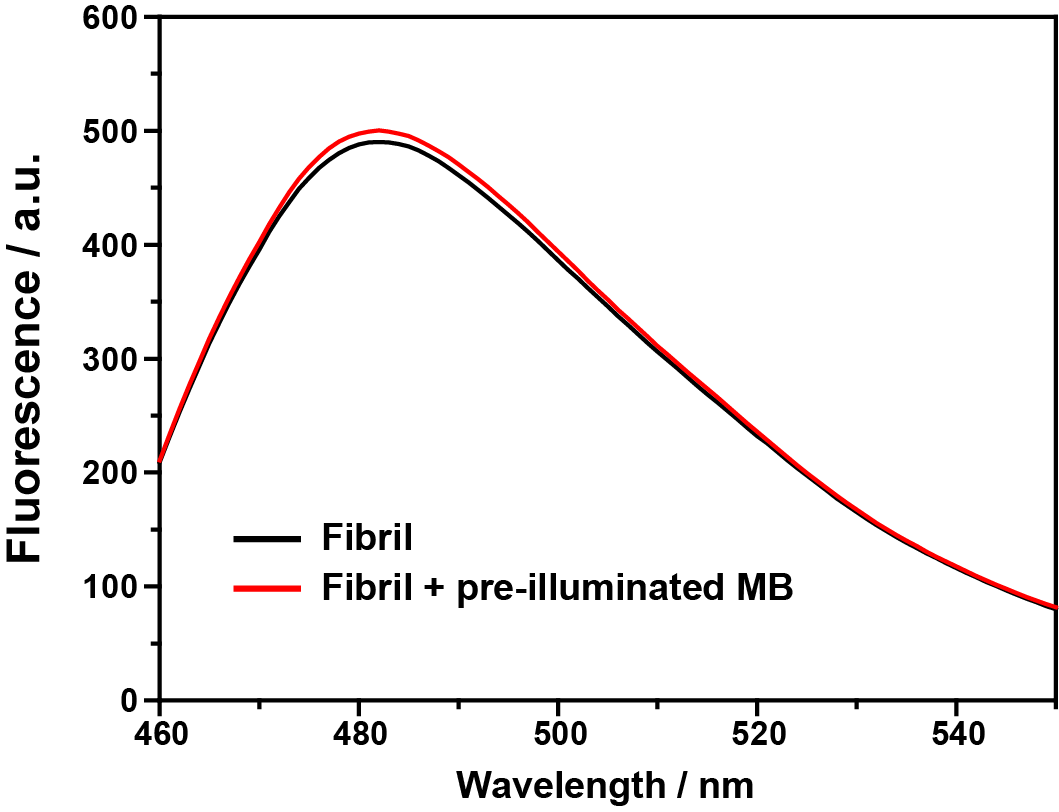


**Figure S2.** ThT assay results of fully aggregated Aβ_42_ measured in the absence and the presence of pre-illuminated MB (10 μM). MB was illuminated for 12 h and introduced right before the fluorescence measurement.


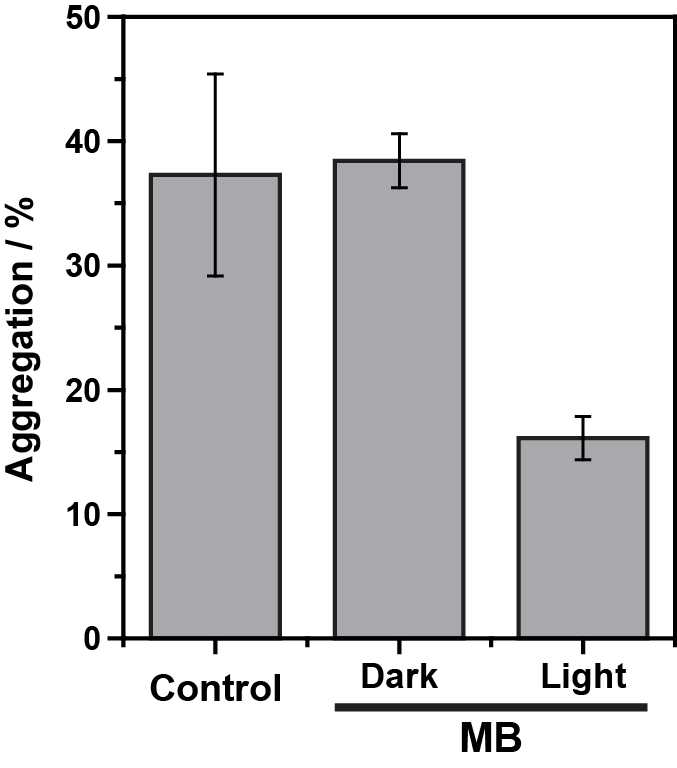


**Figure S3.** The aggregation of MB-treated Aβ_42_ in the absence or presence of light illumination measured with the OD_214_ sedimentation assay. Aβ_42_ (40 μM) was incubated with or without MB (2 μM) under dark or light conditions for 24 h at 30˚C. The aggregation was derived from the OD of Aβ_42_ samples before and after the incubation, recorded at 214 nm.

**
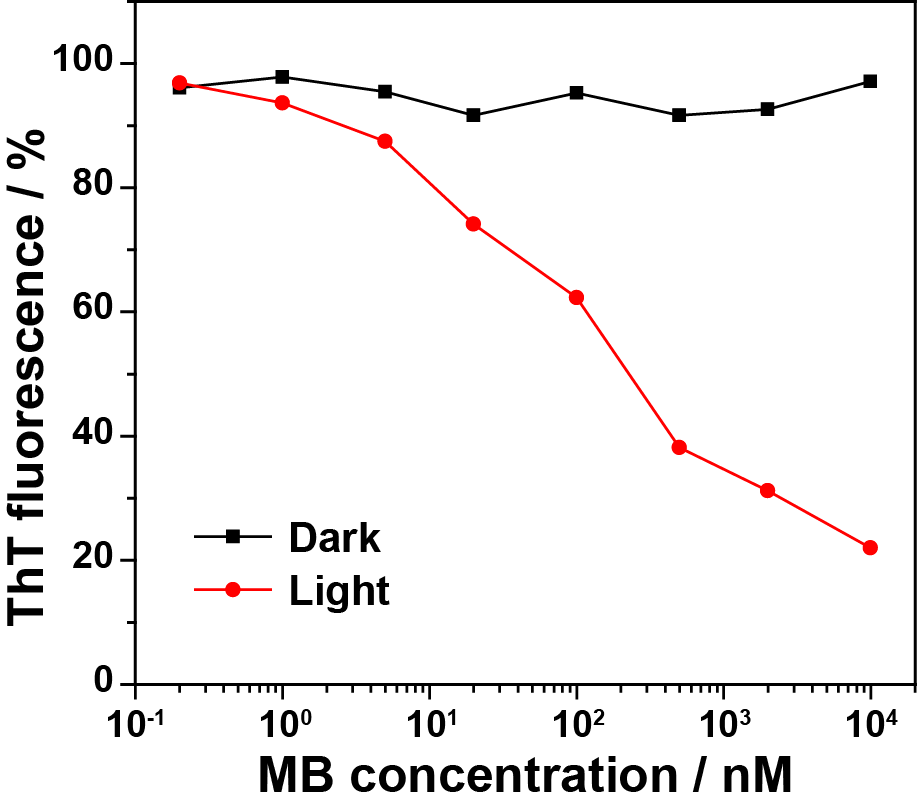
**

**Figure S4.** Concentration-dependent inhibition of Aβ aggregation by MB under dark and light conditions. 40 μM of Aβ was incubated with various concentrations (0.2 nM, 1 nM, 5 nM, 20 nM, 0.1 μM, 0.5 μM, 2 μM, and 10 μM) of MB under dark or light conditions.

**
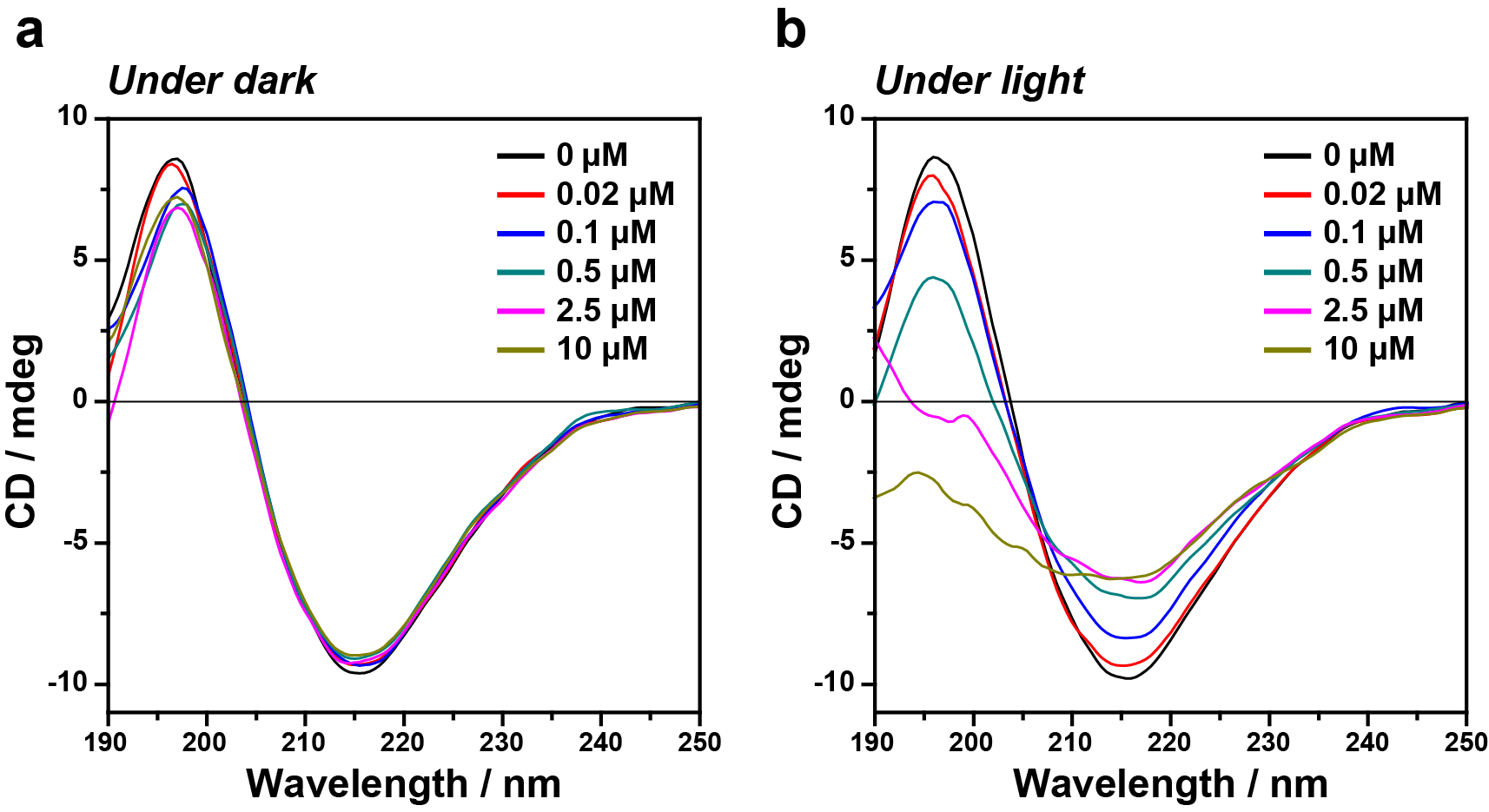
**

**Figure S5.** CD spectra of Aβ incubated with various concentrations (0.02, 0.1, 0.5, 2.5, and 10 μM) of MB under dark and light conditions.


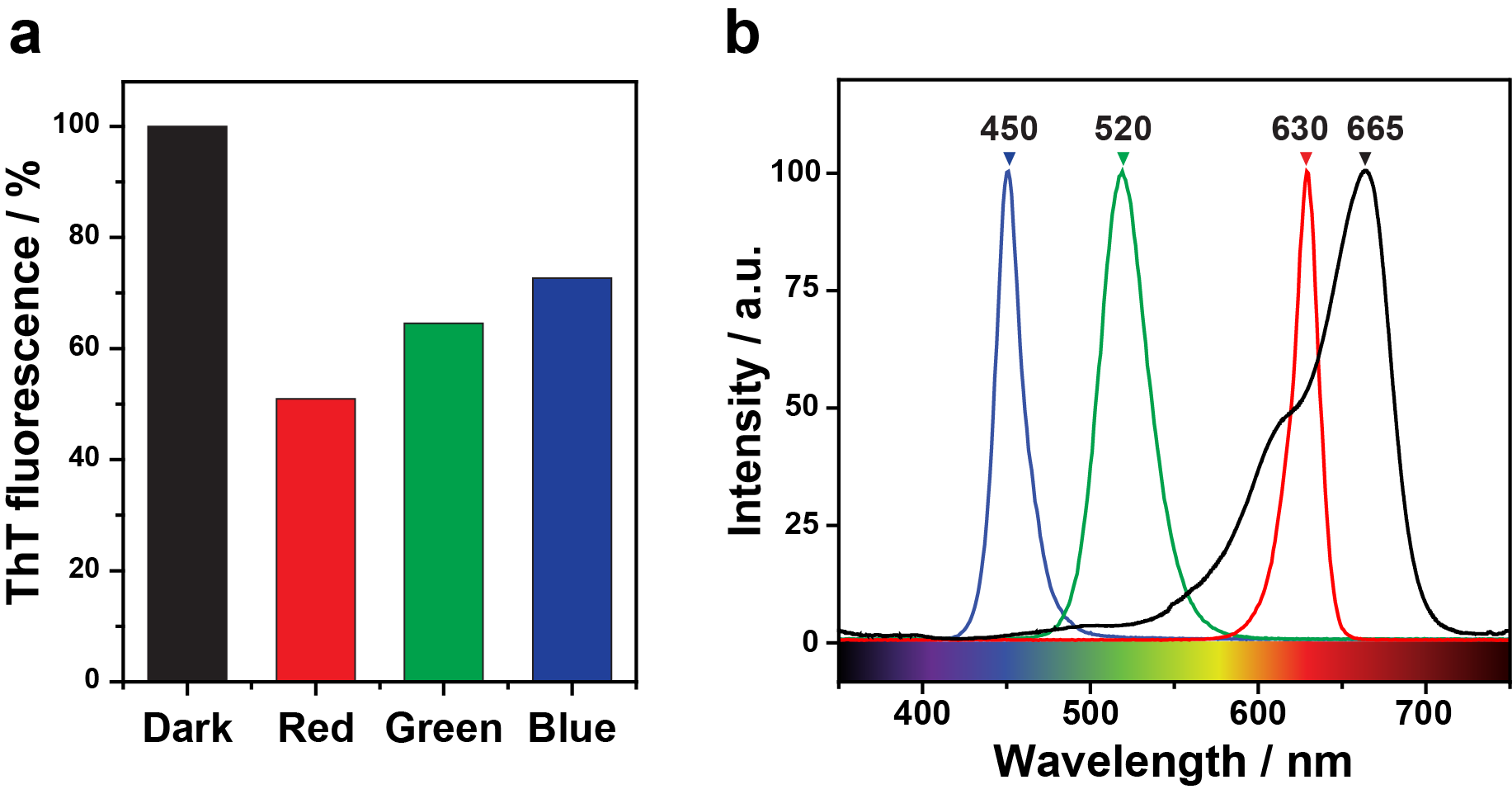


**Figure S6.** Effect of incident light wavelength on Aβ aggregation. (a) Absorption spectrum of MB (black line) in phosphate buffer (pH 8.0) and emission spectra of blue, green, and red LED. The maximum emission wavelengths of LEDs and the maximum absorption wavelength of MB are shown at the top of the graph. (b) ThT fluorescence intensity of Aβ samples treated with 2 μM of MB incubated under dark, red, green, and blue LED lights.

**
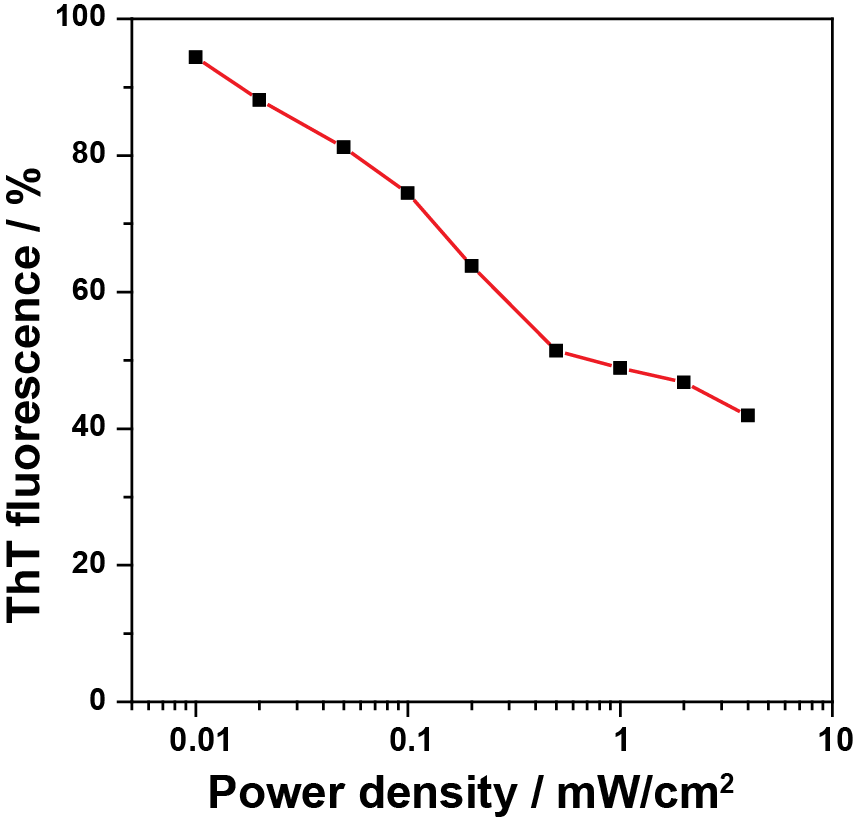
**

**Figure S7.** Effect of power density of light source on Aβ aggregation. The LED power density was increased, while the MB concentration was fixed at 2 μM. The result shows that amyloid aggregate formation decreased with the increasing LED power density (0.01, 0.02, 0.05, 0.1, 0.2, 0.5, 1, 2, and 4 mW/cm^2^).

**
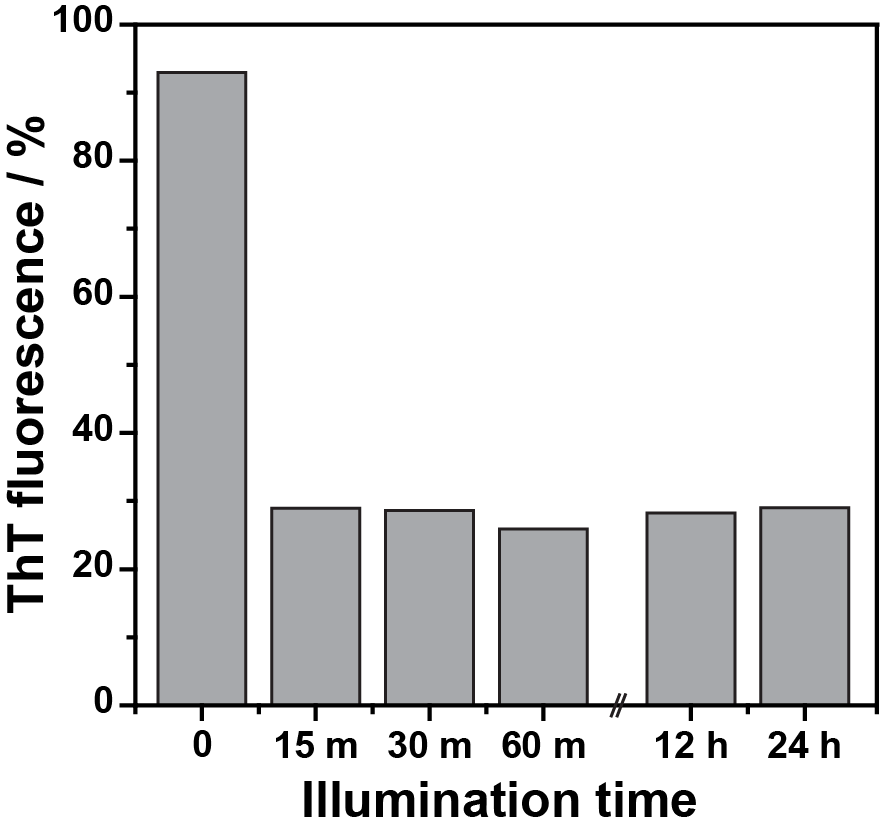
**

**Figure S8.** The effect of light illumination time on the self-assembly of Aβ in the presence of MB. Aβ aggregation was quantified by ThT fluorescence measured at 485 nm with an excitation wavelength of 440 nm.


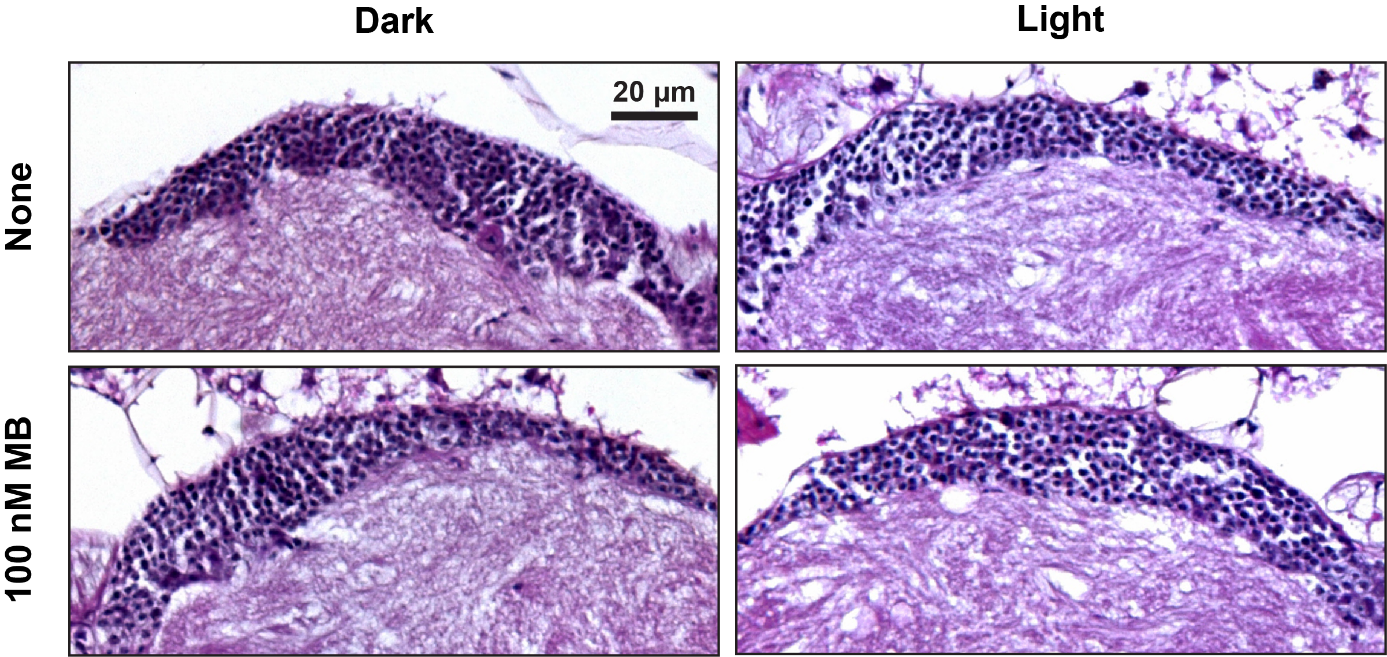


**Figure S9.** Brain section image of the control flies. Representative haematoxylin and eosin staining of adult head sections in control flies (*elav-GAL4/+)* with or without 100 nM MB treatment under dark and red LED light. Scale bar: 20 μm.


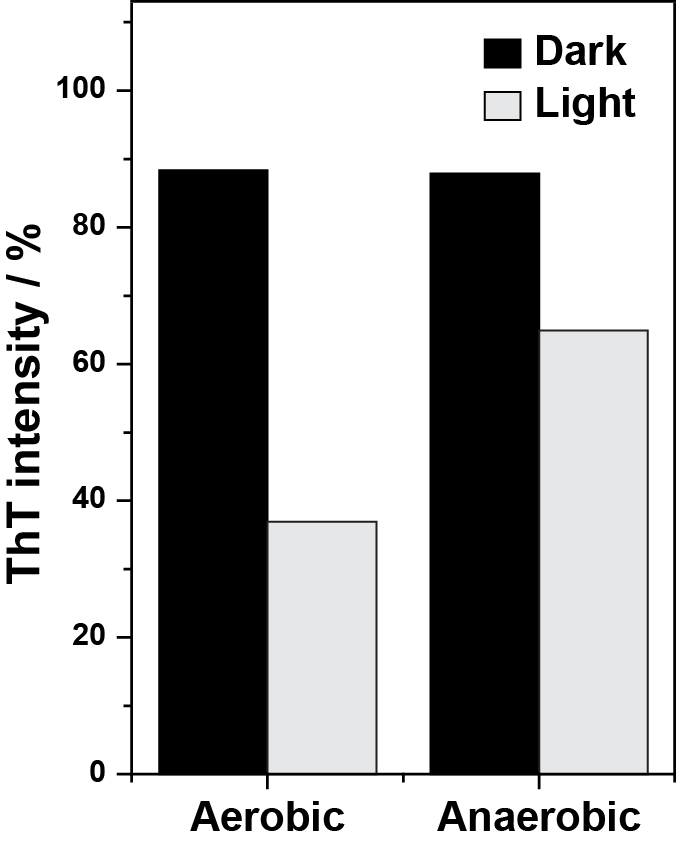


**Figure S10.** The inhibitory activity of MB (0.5 μM) under aerobic and anaerobic conditions. The anaerobic condition was achieved with Ar-purged buffer containing NaN_3_ (2 mM) as a singlet oxygen quencher.


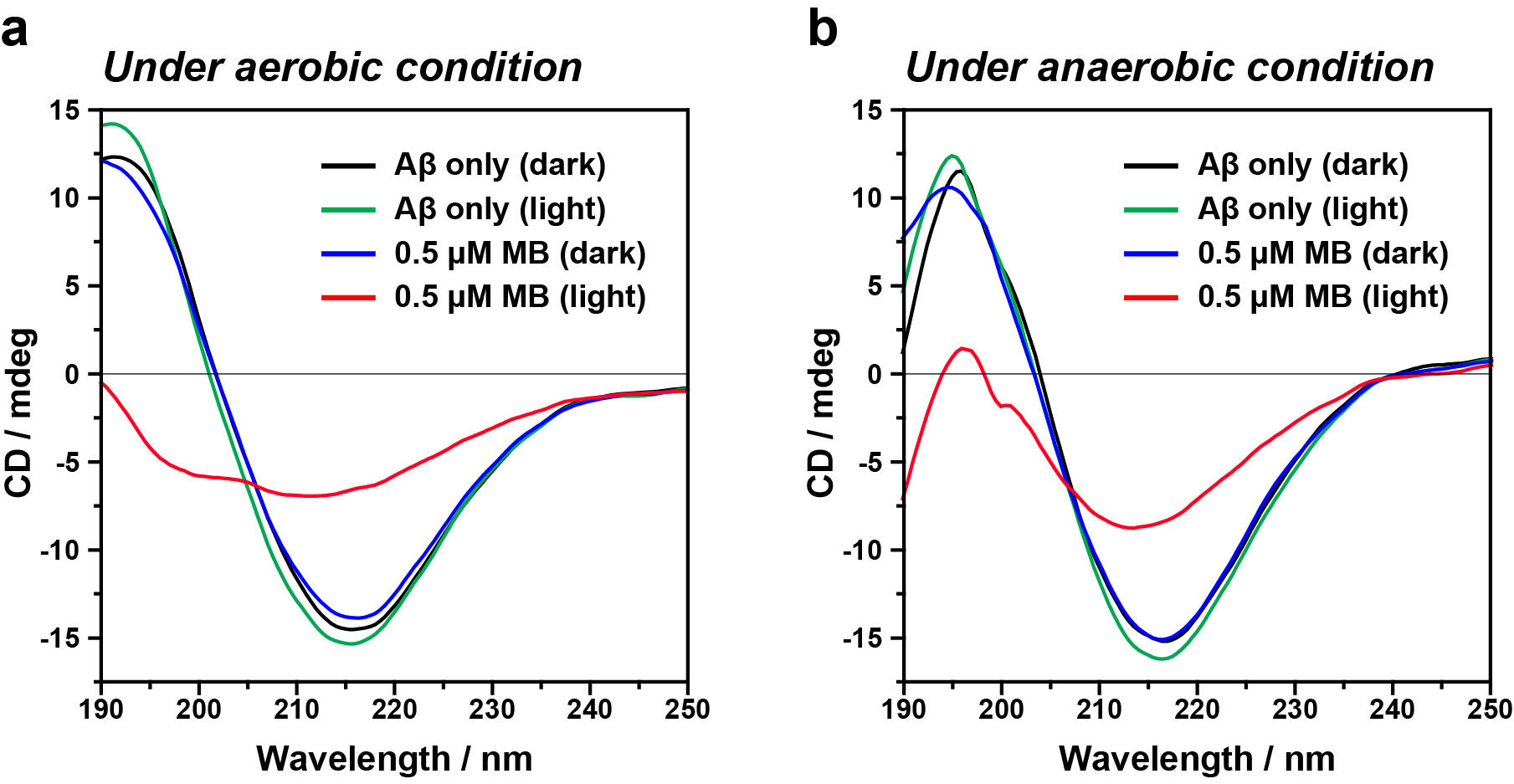


**Figure S11.** Light-induced inhibition by MB under aerobic (a) and anaerobic (b) conditions. The positive peaks at 195 nm had completely disappeared under the aerobic condition (a), while the band remained under the anaerobic condition (b), indicating the suppressed efficacy.


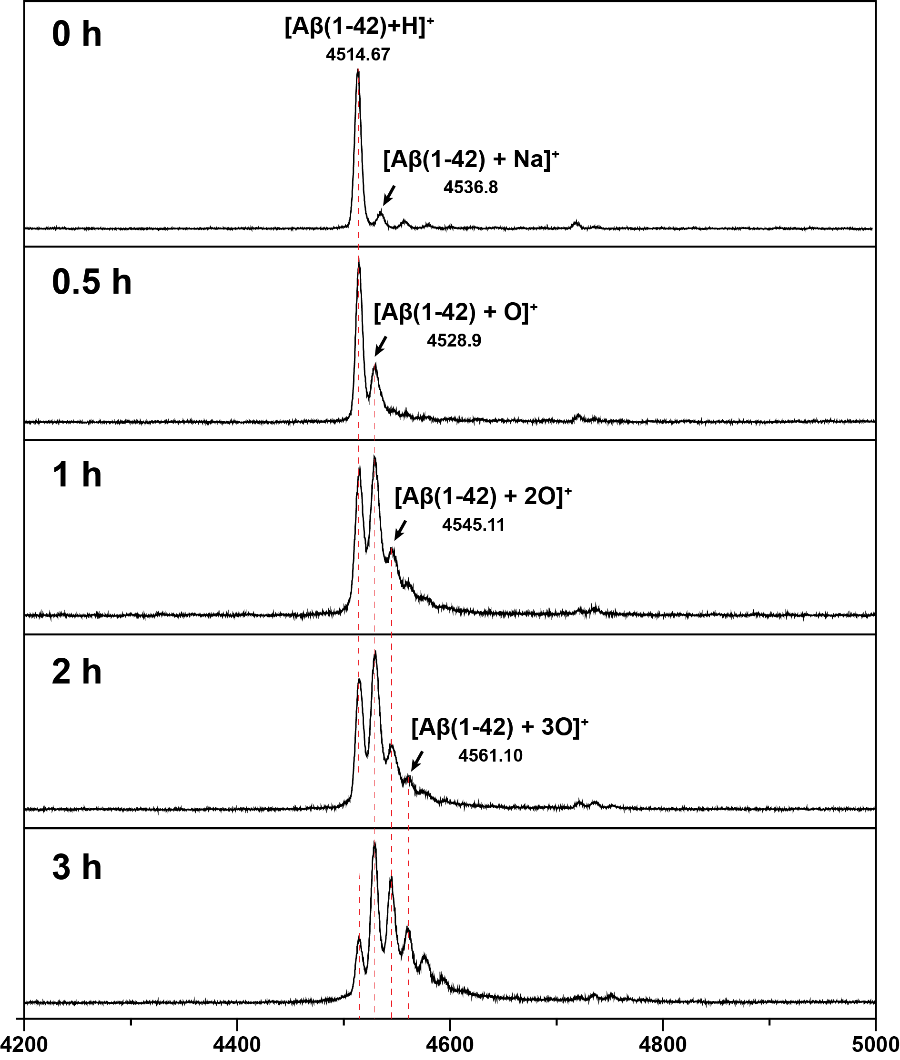


**Figure S12.** MALDI-TOF MS spectra of MB-treated Aβ_42_ irradiated for 0, 0.5, 1, 2, and 3 h. Aβ_42_ (40 μM) samples treated with MB (1 μM) was kept at 4˚C during the light illumination to retain the monomeric contents.


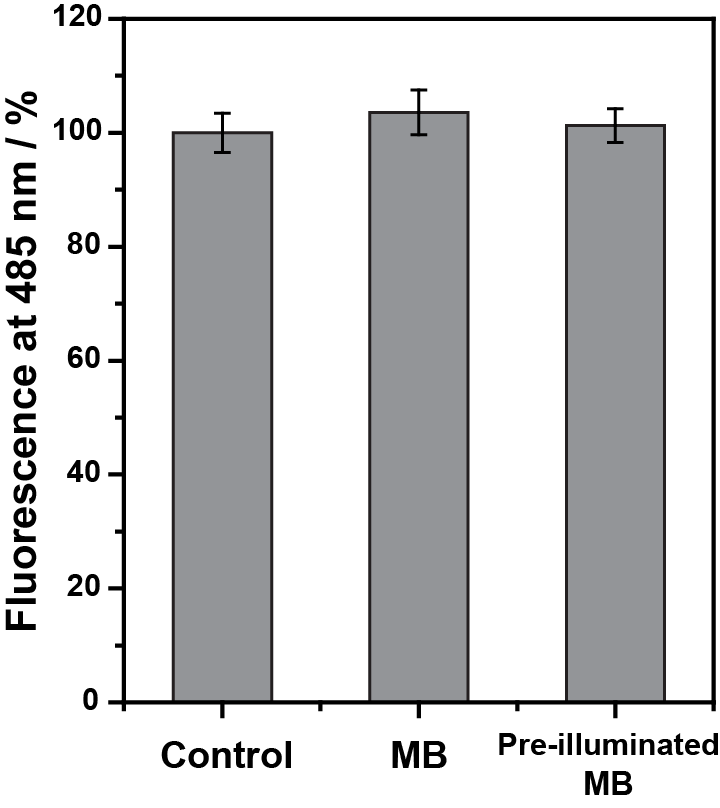


**Figure S13.** ThT fluorescence intensity of Aβ_42_ peptide incubated with MB and pre-illuminated MB for 24 h at 30˚C, under dark. Pre-illuminated MB was irradiated with LED for 12 h prior to the experiment. A negligible decrease in fluorescence implies no inhibitory activity of pre-illuminated MB by itself.
